# Improving Access to Mental Health Care by Delivering Psychotherapeutic Care in the Workplace: A Cross-Sectional Exploratory Trial

**DOI:** 10.1371/journal.pone.0169559

**Published:** 2017-01-05

**Authors:** Eva Rothermund, Reinhold Kilian, Edit Rottler, Dorothea Mayer, Michael Hölzer, Monika A. Rieger, Harald Gündel

**Affiliations:** 1 Department of Psychosomatic Medicine and Psychotherapy, University Hospital Ulm, Ulm, Germany; 2 Department of Psychiatry II, University Hospital Ulm, BKH Günzburg, Germany; 3 Health and Safety, Daimler AG, Sindelfingen, Germany; 4 Sonnenbergklinik, Stuttgart, Germany; 5 Institute for Occupational and Social Medicine and Health Services Research, University Hospital Tübingen, Tübingen, Competence Centre Health Services Research, Medical Faculty Tübingen, Germany; Universitatsklinikum Wurzburg, GERMANY

## Abstract

**Objective:**

Common mental disorders like mood and anxiety disorders and somatoform disorders have high costs, yet under-treatment is still frequent. Many people with common mental disorders are employed, so the workplace is potentially a suitable context in which to provide early treatment. Our study investigates whether a change of setting (workplace versus standard care) improves access to treatment for common mental disorders.

**Methods:**

Conditional latent profile analysis was applied to identify user profiles for work ability (WAI), clinical symptoms like depression (patient health questionnaire depression, PHQ-9), health-related quality of life (QoL, SF-12), and work-related stress (Maslach Burnout Inventory, irritation scale). Patients were recruited consecutively, via psychotherapeutic consultation in the workplace (n = 174) or psychotherapeutic consultation in outpatient care (n = 193).

**Results:**

We identified four user profiles in our model: ‘severe’ (n = 99), ‘moderate I—low QoL’ (n = 88), ‘moderate II—low work ability’ (n = 83), and ‘at risk’ (n = 97). The ‘at risk’ profile encompassed individuals with reduced work ability (36.0, 34.73 to 37.37), only mild clinical symptoms (PHQ-9 5.7, 4.92 to 6.53), no signs of work-related stress and good quality of life. A higher proportion of the ‘at risk’ group than of the ‘severe’ group sought help via the psychotherapeutic consultation in the workplace (OR 0.287, P < 0.01); this effect remained after controlling for gender.

**Conclusions:**

Offering secondary mental health care in the workplace is feasible and accepted by users. Offering treatment in the workplace as an alternative to standard outpatient settings is a viable strategy for improving access to treatment for common mental disorders.

## Introduction

The workplace has been internationally promoted as a pivotal social context to address individuals early in the course of common mental disorders (CMD), like mood and anxiety disorders and somatoform disorders [1–6]. Nevertheless there has been no investigation as to whether changing the context of a mental health care offer to the worksite improves access for individuals with CMD in a mental health care system like Germany. Thus we set out to investigate whether the worksite mental health care offer of “psychotherapeutic consultation in the workplace” (PSIW) [7,8] compared to “psychotherapeutic outpatient care” (PSOC), as a part of the existing comprehensive mental health care system within Germany, improves access for individuals with CMD.

The treatment gap for CMD, i.e. between individuals requiring care for mental ill-health and those who finally receive mental health care, has been estimated globally at 55% [9,10]. The barriers to mental health care are diverse [5]: On the one hand, they often involve a combination of insufficient resources and inadequate health policies. On the other hand, specialist services are often not utilised by affected individuals [11,12] because of the fear of stigmatisation [13–15] or because of gender role expectations [16,17]. In particular, men report remarkably low and delayed utilisation rates for mental health care [18,19].

Mental ill-health causes personal suffering, reduced quality of life, and reduced employment prospects. Furthermore CMD are the focus because mental health problems are a strong predictor for future impaired work functioning and negative clinical outcomes [20,21]. Indeed, CMD constitute a leading cause of absenteeism and early retirement in Europe, and they result in remarkable and rising direct and indirect costs in industrial countries [10,22]. Chronic CMD usually requires higher levels of treatment [23–25]. Therefore, in recent times, the urgent need for early intervention has been outlined [6]. Nevertheless, there is often a delay in initial treatment [9,10]. That delay contributes to the risk of chronicity [26,27], decreased job performance [28], future sickness absences, and even early retirement [29]. As about 15% of the working-age population experiences CMD, and many of them are not yet on sickness absence, a significant number of people with mental health problems are employed and at their workplaces [4,10]. That explains why the workplace has been promoted as a pivotal social context in which mental health problems should be addressed and treated early [1,6].

In the study context of the German healthcare system, patients with CMD are treated with psychotherapy by physicians specialising in psychiatry or psychosomatic medicine or by psychological psychotherapists. Treatment is usually delivered through private practices, the outpatient clinics of psychosomatic hospitals and psychosomatic departments or psychosomatic outpatient clinics at general hospitals [30]. In the German healthcare system, PSOC is covered by statutory health insurance as well as by private health insurance, and nearly 100% of the population is covered by health insurance. Thus comprehensive care should be available to all those who need it. In spite of this, the treatment gap for CMD in Germany is comparable to that of other European countries [31].

Thus the present study examined if the worksite intervention (PSIW) improves access for individuals with CMD compared to the standard care offer (PSOC). Improved access was defined as contact with the mental health care offer (PSIW or PSOC) in an early state of impairment. To identify different subgroups of disease or impairment severity in PSIW and PSOC we applied the instrument of latent profile analysis (LPA).

It enabled us to identify meaningful, homogeneous subgroups of individuals within the heterogeneous group of all users of the services. LPA is a method of classifying individuals into distinct groups based on individual response patterns on several characteristics [32]. Therefore it was possible to include work ability, mental health measures, QoL and work-related stress measures to get a more differentiated picture of how CMD impacts a person’s life.

## Materials and Methods

### Study design

An observational cross-sectional design was chosen to explore the user profile of the new care model, PSIW, as compared to PSOC. It was part of a mixed-methods study [33], German Clinical Trials Register (DRKS) trial registration number: DRKS00003184. The study was retrospectively registered two and a half month after the first patient recruitment on 13 January 2012 due to a delay in the registration process. Based on the research question: “Do we reach different users by changing the context?”, LPA was applied to identify subgroups of users with regard to their response patterns to the following measures: work ability (WAI), clinical symptoms (PHQ), health-related quality of life (QoL, SF-12) and work-related stress (MBI, IS). Using conditional latent profile analysis, the relation of each subgroup to the respective treatment setting, i.e. workplace (PSIW) or psychotherapeutic outpatient care (PSOC as part of standard care in Germany) could be analysed i.e. whether a certain profile could be predictive for the use of the type of psychotherapeutic service (PSIW or PSOC).

**Fig 1:**
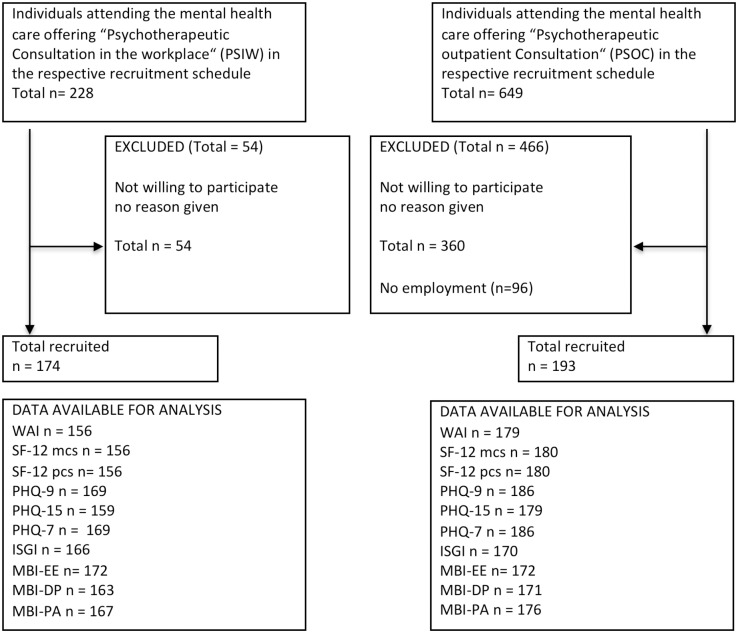
Flowchart participants according to the STROBE criteria [34].

The patients who participated gave informed written consent before they answered self-administered questionnaires prior to the initial consultation. Symptom duration was explored and documented by the psychotherapist after the consultation. The study is reported according to the STROBE criteria [34] (Fig 1). Ethical approval was given by the Medical Ethical Board of the University Medical Centre of the University of Ulm (26th September 2011) and the study was conducted according to principles expressed in the Declaration of Helsinki.

### Setting

Psychotherapeutic consultation, as a secondary mental health care offering, was investigated either in the workplace (PSIW) with three participating companies or as part of the standard care (PSOC) with two participating psychosomatic outpatient clinics in Southwest Germany.

The intervention in both groups (PSIW and PSOC) included an initial consultation, a diagnostic assessment, an indication, crisis intervention (if needed), as well as support for a referral to the existing secondary mental health care system. The intervention was performed by a psychotherapist, i.e. a medical doctor (psychiatric or psychosomatic specialisation) or psychologist specialising in psychotherapy. In the case of PSIW, the intervention was part of and located within the company health care promotion. In contrast to PSIW, PSOC was delivered as standard psychotherapeutic outpatient care.

### Participants

To be eligible, participants had to be at least 18 years old and capable of understanding the German language in its spoken and written forms.

Participants in the PSOC group were recruited consecutively from two outpatient clinics: University Clinic of Psychosomatic Medicine and Psychotherapy, Ulm, and Sonnenbergklinik, Division of Psychosomatic Medicine of the ZfP, Suedwuerttemberg, Stuttgart (06/2012-01/2013). These patients were mainly referred by general practitioners, although they were partly self-referred. In the PSOC group, individuals without employment were excluded before the data analysis (Fig 1).

Participants in the PSIW group had to be employed by one of the participating companies offering PSIW and were recruited consecutively from 11/2011–06/2013 in three companies: an automobile manufacturer, a metal works company, and a security systems company. The employees in two companies were mainly referred to PSIW by the occupational physicians or the social workers. The employees at the third company were mainly self-referred.

Due to protocol restrictions, reasons for non-participation or any information about non-participants were not recorded. Only in the case of the non-participants of PSOC of the University Clinic of Psychosomatic Medicine and Psychotherapy were we able to compare age and gender of participants (n = 162) and non-participants (n = 394). As reported in Table 1, no differences in these parameters were detected.

**Table 1:** Non-participant analysis in a subgroup of PSOC.

	Participants (n = 162)	Non-Participants (n = 394)	P	Test
Age (in years)	39.8 (SD 11.97)	39.4 (SD 14.42)	n.s.	t-test
Gender (male/all)	55/162	143/394	n.s.	Chi-square test

### Measures

#### Utilisation and symptom duration

Previous psychotherapeutic treatments (lifetime) and previous contacts with the mental health care system (12-month-prevalence) were documented. Symptom duration with respect to the reason for the current referral was assessed by the therapist in months.

#### Work ability

The work ability index (WAI, short version) is a self-referred instrument used to assess current and future work ability, as well as work demand management, based on behavioural measures [35].

#### Mental health and somatic symptoms: depression, anxiety, somatoform symptom severity

Depression was assessed with the 9-item patient health questionnaire depression scale (PHQ-9) [36], anxiety with the 7-item patient health questionnaire generalised anxiety disorder scale (PHQ-7) [37], and somatic symptom severity with the 15-item patient health questionnaire somatisation (PHQ-15) [38]. The interpretation of the PHQ is based on the diagnostic criteria of the DSM-IV and ICD-10 with a recommended cut-off of 10 or above for distinguishing between clinical and non-clinical populations [39].

#### Health-related quality of life: SF-12

The SF-12 is the validated short version of the SF-36, an instrument that measures the functional health status of patients [40]. Weighted summation provided summary scores for perceived mental health (MCS = mental health component score) and perceived physical health (PCS = physical health component score).

#### Work-related strain

Burnout: Maslach Burnout Inventory (MBI), German general version (MBI-GS-D). The MBI is used to assess burnout syndrome complaints as a manifestation of mental exhaustion [41]. Although it overlaps with the depressive syndrome (ICD-10), it is a useful workplace mental health cause-and-effect model. The three components of burnout are: emotional exhaustion (EE), depersonalisation (DP), and (reduced) personal accomplishment (PA). The items are classified according to frequency. To act in line with the other indicators in the model, sum scores instead of means were used for each subscale.

#### Irritation: The irritation scale (IS)

Irritation is defined as subjectively perceived emotional and cognitive strain in occupational contexts [42,43].

### Bias

A random assignment of study participants was not suitable because the assessment of acceptance of the workplace consultation program was a main subject of the study. Confounding bias was statistically controlled by means of multivariate statistical methods.

### Analysis

**Fig 2:**
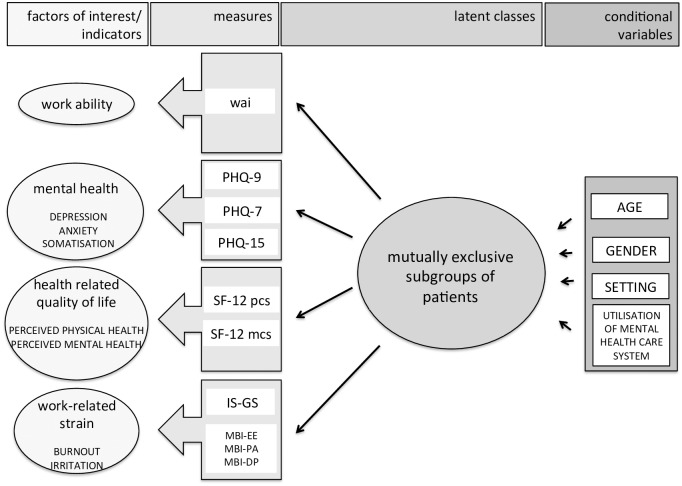
Conditional latent profile model. Factors of interest as continuous variables. Measures by the instruments WAI = work ability index, PHQ-9 Patient health questionaire 9 Items—depression, PHQ-7 Patient health questionaire 7 Items—anxiety, PHQ-15 Patient health questionaire 15 Items—somatoform symptom severity, SF12 = Health related quality of life, pcs = physical component score, mcs = mental component score, IS-GS = Irritations scale, global score, MBI = Maslach Burnout Inventory, EE = emotional exhaustion, PA = personal accomplishment, DP = depersonalisation.

The method of conditional latent profile analysis (CLPA) was chosen to identify groups with different patterns of clinical characteristics and the association between group membership and setting (PSIW or PSOC) (Fig 2). LPA allowed the prediction of latent class membership by a set of covariates labelled as indicators in Fig 2. The continuous measures in our study were normally distributed. The probability of conditional latent class membership was estimated by means of an alternating maximum likelihood approach using the expectation-maximisation (EM) algorithm [32,44]. The number of latent classes was determined via the Bayesian Information Criterion (BIC), the Akaike Information Criterion (AIC), and the bootstrap likelihood ratio test (BLRT) [44,45]. The effects of the covariates on the probability of latent class membership (see Fig 2) were estimated by means of a multinomial logit model [32].

The covariates for the prediction of latent class membership were the respective treatment settings, age, gender, and previous (12-month) utilisation of the mental health care system.

Data coding and descriptive statistics were performed with SPSS version 21. Latent class analysis was computed using Mplus version 7.1 [46].

#### Sample size

For the reported topic, the number of users of each service who were willing to participate during the study period determined the sample size. As a follow-up was part of the study, statistical power analyses were conducted with regard to the WAI as a primary outcome [33], while taking into consideration the sample size requirements for LPA as described by Tein et al [44].

## Results

Three hundred and sixty-seven participants were included (patient flow Fig 1, sample description Table 2). N = 174 constituted the PSIW group, and n = 193 the PSOC group. The PSIW group was older (45 years, SD 10.1) than the PSOC group (40 years, SD 12.1). Symptom duration tended to be shorter (38 months, SD 65.4) in PSIW than in PSOC (51 months, SD 72.9). While in PSIW, there were 122 men (70% of all PSIW users), in PSOC, 66 men participated (34% of all PSOC users). Utilisation of the mental health care system within the previous 12 months was lower in users of PSIW (n = 65) compared to PSOC (n = 119).

**Table 2:** Sample description.

Characteristics		Total sample (n = 367)	PSIW (n = 174)	PSOC (n = 193)	P value
Age (years)	mean (SD)	42.94 (11.47)	45.20 (10.12)	40.05 (12.07)	*** b
Symptom duration (months)	mean (SD)	44.94 (69.68)	38.02 (65.41)	51.06 (72.87)	n.s. b
Gender (male)	n (%)	188 (51.2)	122 (70.1)	66 (34.2)	*** a
Living in steady relationship (yes)	n (%)	239 (68.5)	126 (75.9)	113 (61.7)	* a
Education level	n (%)	364 (100)	171 (100)	193 (100)	
Not finished		4 (1.1)	1 (0.6)	3 (1.6)	n.s. a
Low		106 (29.1)	57 (33.3)	49 (25.4)	
Medium		133 (36.6)	55 (32.2)	78 (40.4)	
High		121 (33.2)	58 (33.9)	63 (32.6)	
First time user (no in- or outpatient treatment ever)	n (%)	185 (54.1)	102 (65.4)	83 (44.6)	***a
Utilisation mental health care system “yes” (12-months)	n (%)	184 (50.1)	65 (37.4)	119 (61.7)	***a

* P < 0.05,

** P < 0.01,

*** P < 0.001,

SD = standard deviation, n = number, a = chi-square test, b = t-test

### Conditional latent profile analysis

The goodness-of-fit indices for three to five class models are presented in Table 3. Based on fit indices and interpretability of class solutions, a four-class solution was judged to be the optimal solution. This solution comprises four profiles: a “severe” profile, a “moderate affected I-low QoL” profile, a “moderate affected II-low WAI” profile and an “at risk” profile (see Table 4).

**Table 3:** Goodness-of-fit statistics for 3 to 5 class solutions.

Model tested, distribution of individuals	AIC	BIC	aBIC	Entropy	BTRL P-value
3 Classes 112/166/89	21,993.526	22,196.604	22,031.628	0.851	0.000
4 Classes 99/88/83/97	21,908.272	22,173.837	21,958.098	0.827	0.000
5 Classes 80/89/82/71/45	21,824.234	22,152.284	21,885.784	0.822	0.000

**Table 4:** Latent profiles of impairment for four-class solution.

Profile label/ mnemonic N (class probability) all n = 367 (100%)	“severe” (profile 1) n = 99 (27%)		“mod.I-low QoL” (profile 2) n = 88 (24%)		“mod.II-low WAI” (profile 3) n = 83 (23%)		“at risk” (profile 4) n = 97 (26%)	
measure	Mean	95% CI	Mean	95% CI	Mean	95% CI	Mean	95% CI
WAI	18.6	16.31 to 20.94	30.5	27.90 to 33.13	23.4	21.54 to 25.29	36	34.73 to 37.37
SF-12 mcs	23.7	22.25 to 25.24	25.9	22.56 to 29.15	32.2	30.27 to 34.20	44.1	41.36 to 46.80
SF-12 pcs	38.8	35.05 to 42.49	53.2	50.42 to 55.95	35.1	31.86 to 38.25	50.8	48.80 to 52.88
PHQ-9-depr	19.3	17.95 to 20.67	14.1	11.74 to 16.53	12.5	11.07 to 14.02	5.7	4.92 to 6.53
PHQ-15-so	15.6	13.89 to 17.28	9.6	8.40 to 10.90	12.6	11.34 to 13.91	6.5	5.70 to 7.39
PHQ-7-anx	15.3	14.10 to 16.57	11.7	9.75 to 13.64	9.8	8.43 to 11.23	5	4.18 to 5.91
IS-GS	43.6	41.33 to 45.91	36.6	32.60 to 40.69	32.4	29.35 to 35.45	20.6	18.13 to 23.00
MBI-EE	26.7	25.86 to 27.52	22	20.17 to 23.79	22.2	20.85 to 23.61	13.9	12.37 to 15.38
MBI-DP	21.2	20.04 to 22.44	18.5	16.47 to 20.53	15.4	13.09 to 17.76	11.6	10.25 to 12.91
MBI-PA	23.1	21.75 to 24.50	26.1	24.38 to 27.82	27.3	25.62 to 28.98	29.7	28.81 to 30.56
Measure	Instrument	Range	References/interpretation				Source	
WAI	work ability index	7–49	49–44 very good work ability	43–37 good work ability	36–28 moderate work ability	27–7 very low work ability	[35]	
SF-12-mcs	SF-12-mental component score	0–100	compared with normative German sample 1994: 51.2, psychosomatic inpatients 27				[40]	
SF-12-pcs	SF-12-physical component score	0–100	to compare: normative German sample 1994: 46.3, psychosomatic inpatients 40					
PHQ-9-depr	patient health questionnaire depression	0–27	0 to 4 minimal symptom burden	5 to 9 mild symptom burden	10 to 14 moderate symptom burden	> 15 severe symptom burden	[39]	
PHQ-15-som	patient health questionnaire somatoform symptom severity	0–30						
PHQ-7-anx	patient health questionnaire anxiety	0–21						
ISGI	irritation sale global index	8–56	no irritation 8–16	low irritation 17–26	moderate irritation 27–37	strong irritation 38–56	[42]	
MBI-EE	burnout-emotional exhaustion	5–30	the lower the healthier				[41]	
MBI-DP	burnout- depersonalisation	5–30	the lower the healthier					
MBI-PA	burnout—personal accomplishment	6–36	the higher the healthier					

While a five-class solution yielded marginally lower AIC, BIC, and aBIC indices than the four-class solution, the entropy value was lower (Table 3). The average latent class probabilities for most likely latent class membership were better for the three-class solution (0.922–0.964), but still very good for the four-class solution (0.860–0.968). Discriminative propriety was satisfied, as mean class probability to another class membership stayed under 0.10. The BLRT was still significant for the four class solution, indicating that a five-class solution would provide additional information. However, since the latent profile of the additional fifth class was not distinguishable from the other classes, the more parsimonious four-class solution was retained.

### Latent profiles emerging from the model

We used LPA to investigate heterogeneity. Four subgroups of patients could be identified in the respective treatment settings. The “severe” profile showed the worst scores with regard to work ability, mental health, QoL, and work-related stress. Both moderate profiles were moderately affected with regard to common mental health and already showed alerting scores for work-related stress. Whereas the “moderate I—low QoL” profile was especially affected by low QoL with regard to mental health, the profile “moderate II—low WAI” showed quite good QoL, but reported a work ability as low as those in the “severe” profile. Users with the “at risk” profile reported only mild disturbance in mental health, QoL, and work-related stress.

The overall means for the indicators (Fig 2) and the respective confidence intervals are reported in Table 4. Descriptive data for the four profiles are reported in Table 5.

**Table 5:** Descriptive data of the four profiles.

	“severe” (profile 1)	“mod.I-low QoL” (profile 2)	“mod.II-low WAI” (profile 3)	“at risk” (profile 4)
Class probability n	99	88	83	97
Age (SD)	43.4 (10.5)	40.4 (11.9)	46.5 (10.5)	40.0 (11.9)
Gender (female, %)	50.5	52.3	50.6	42.3
Setting (PSIW, %)	30.3	46.6	49.4	63.9
Utilisation* (%)	74.7	37.5	56.6	30.9

* utilisation mental health care system “yes” previous 12-months

Profile 1 (“severe”) was comprised of 99 patients with results indicating great impairment over all measures and compared to the other subgroups (Table 4). In detail, they reported very low work ability (18.6, 16.31–20.94) and very low scores in perceived mental (23.7, 22.25 to 25.24) and physical QoL (38.8, 35.05 to 42.49). The scores in the PHQ indicated that these patients evidenced severe, with a high probability of clinically significant, symptoms of CMD, i.e. depression, anxiety and somatoform symptom severity. Highly expressed work-related stress (IS 43.6, 41.33 to 45.91, MBI-EE 26.7, 25.86 to 27.52) was reported by individuals in this group.

Profile 2 (“moderate affected I-low QoL”) was comprised of 88 participants with moderate affected work ability (30.5, 27.90 to 33.13) and moderate expressed CMD (depression, anxiety, and somatoform symptom severity): From a clinical perspective, these individuals were likely to be diagnosed and to require treatment. Patients in the profile “moderate I-low QoL” experienced a very high level of suffering in daily life, corresponding to a low score of SF-12 mcs (25.9, 22.56 to 29.15), comparable to those in the “severe” profile. In contrast to the “moderate II-low WAI” profile, they reported good physical health (53.2, 50.42 to 55.95, SF-12 pcs). Individuals in the “moderate I” profile showed alerting scores for work-related stress (IS 36.6, 32.60 to 40.69, MBI-EE: 22.0, 20.17 to 23.79).

Profile 3 (“moderate affected II-low WAI”) accounted for 83 individuals with seriously impaired work ability (as bad as in the severe group) (23.4, 21.54 to 25.29). Physically, they were not that badly affected (35.1, 31.86 to 38.25, SF-12pcs), but they scored in the risky area for psychological daily impairment (32.2, 30.27 to 34.20, SF-12mcs). Meanwhile, individuals in this group experienced moderate clinical affection similar to those with the “moderate-I-low QoL,” profile i.e.: From a clinical perspective, they were likely to be diagnosed with CMD and to require treatment. Individuals in this group showed alerting scores for work-related stress (IS 32.4, 29.35 to 35.45, and MBI-EE 22.2, 20.85 to 23.61).

Profile 4 (“at risk”): Reduced work ability (36.0, 34.73 to 37.37) was the most alerting assessment for the 97 individuals in this group. They were mildly affected from the clinical point of view. Mild psychological impairment (44.1, 41.36 to 46.80, SF-12 mcs) could already be detected. Neither physical impairment (50.8, 48.80 to 52.88, SF-12 pcs) nor burnout (13.9, 12.37 to 15.38, mbi-EE) could be detected. Irritation (20.6, 18.13 to 23.00) indicated incipient risk.

### Predictors of profile membership

The results of the multinomial logit regression model (see Table 6) revealed that, compared to those in the “at risk” profile, individuals in the “severe” profile were more likely to be older (OR 1.037, p<0.05), to show up in regular care (OR 0.287, p<0.01), and to report higher utilisation of the mental health care system within the previous 12 months (OR 4.427, p<0.001). Compared to those in the “at risk” profile, individuals in the “moderate II-low WAI” profile appeared to be older (OR 1.065, p<0.001). No further differences could be detected, i.e. compared to those in the “at risk” profile, individuals in the “moderate I” profile were comparable with regard to age, gender, setting, and utilisation.

**Table 6:** Multinomial logistic regression predicting class membership.

	OR	SE	P value
Profile 1 severe (vs. profile 4 “at risk”)			
Age	1.037	0.017	*0*.*032*
Gender = female	1.101	0.372	0.795
Setting = PSIW	0.287	0.411	*0*.*002*
Utilisation*	4.427	0.374	*0*.*000*
Profile 2 moderate I-low QoL (vs. profile 4 “at risk”)			
Age	1.012	0.017	0.464
Gender = female	1.202	0.378	0.626
Setting = PSIW	0.542	0.412	0.136
Utilisation*	1.116	0.376	0.771
Profile 3 moderate II-low WAI (vs. profile 4 “at risk”)			
Age	1.065	0.018	*0*.*000*
Gender = female	1.347	0.379	0.432
Setting = PSIW	0.504	0.414	0.098
Utilisation*	2.125	0.396	0.057

SE = Standard Error, OR = Odds Ratio, P value = level of significance,

* utilisation mental health care system “yes” previous 12-months

## Discussion

This cross sectional study revealed strong evidence that changing the context of a mental health intervention from standard care into the vocational setting attracts more users with lower overall impairment than in established outpatient care. To identify differing user profiles with regard to mental impairment and their relation to the respective treatment setting, the instrument of conditional latent profile analysis was applied. Four user profiles: ‘severe’, ‘moderately affected with low QoL’, ‘moderately affected with low work ability’, and ‘at risk’ were identified. A higher proportion of the ‘at risk’ group than of the ‘severe’ group sought help via the workplace. The results demonstrate that the workplace constitutes a setting in which individuals with CMD can be successfully addressed with a view to early intervention.

**Fig 3:**
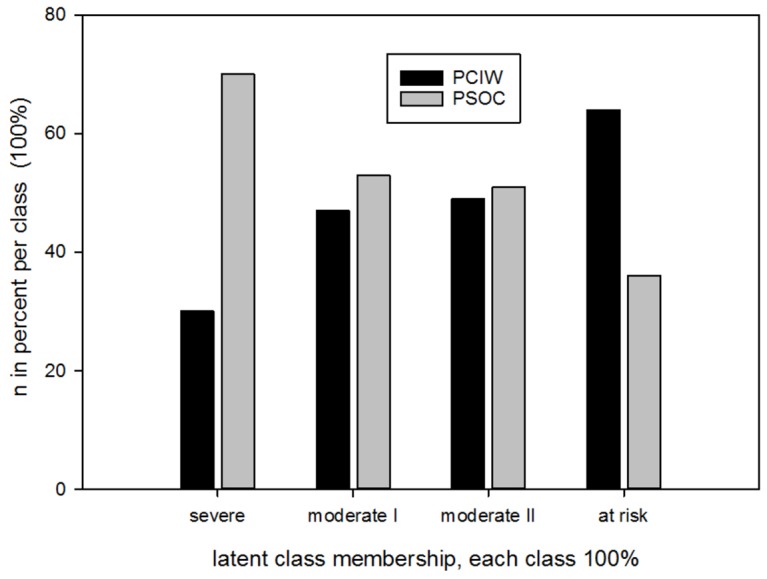
Distribution of different user groups in the respective treatment settings. Severe (n = 99), moderate I (n = 88), moderate II (n = 83), at risk (n = 97)

Implementing a new secondary care offering in the setting of the workplace gives rise to the question of acceptance, especially due to the fear of stigmatisation and stereotypic gender roles [5,13–17]. According to male utilisation rates of traditional mental health care offerings, 30% of men in our study made use of standard psychosomatic outpatient care (PSOC). Contrary to this finding, 70% of users of PSIW were male (in line with the demographics of the companies) [18,19]. The gender distribution was similar for the four distinct user profiles, and all profiles were distributed over both the vocational and the standard outpatient setting (Tables 5 and 6, Fig 3). Thus, it can be concluded that acceptance of the new mental health care service was high, and fear of stigmatisation possibly played a minor role.

The “at risk” group showed impaired work ability even though their clinical impairment was only mild. That is in agreement with the findings of Bertilsson et al [47], who described work capacity as an early predictor for future sickness absence. Previous research revealed that even mild depressive impairment is likely to reduce work productivity [48]. Thus, we conclude that in the “at risk” group, we have identified individuals with a high risk of future sickness absence, who could benefit from early intervention. Previous findings have identified interpersonal problems at the workplace as one of the main drivers for mental discomfort in actual work environments [49–51].

Moreover, our data show that the four distinct user profiles follow differential pathways to mental health care offerings. Whereas the “severe” profile was mainly observed in PSOC (70%), the “at risk” profile predominately made use of the offering within the vocational context (64%) (PSIW) (Fig 3). These findings clearly demonstrate a high potential to reach a group at an early stage of a mental disorder (“at risk” profile) with the offering in the vocational context.

It is widely accepted that mental disorders should be treated during the early stages of onset [6], as they are known to be especially responsive to treatment during the early phases of illness, e.g. as demonstrated by dell´Osso et al [27]. Early intervention has been shown to be successful in outpatient settings [5,52–54] and as a worksite intervention [5,55]. Thus, a recommendation to address patients early in the course of disease has been drafted. Likewise, key European and international organisations, practitioners, and policy makers have highlighted the workplace, both at a policy and practice level, as an important setting to address mental health problems for early intervention even in countries with comprehensive mental care for large parts of the population [1–6]. Our data clearly provide strong evidence for this concept.

However, some limitations are worth noting. First, the measures used in the analysis were patient-reported outcome measures (PRO). Thus, unfortunately, we are not able to provide expert validated information whether patients with severe mental illness were among the ‘severe’ group or not. Self-reported measures in mental health and work studies are widespread, but are often combined with an expert rating or structured interview [49,56,57]. Nevertheless, some important investigations concerning mental health worked with PRO [58,59], especially in investigating work ability, e.g. Ferrie et al [60] have provided evidence that PRO can be used successfully. A second limitation was the recruitment within companies and in psychotherapeutic outpatient clinics: Although the participation rate was high, not enough patients could be recruited within the foreseen time schedule. For this reason, we added a third company and extended the time schedule for recruitment. The high rate of non-participation in the PSOC group could be analysed partly and did not show differences between participants and non-participants due to age and gender. For this reason, and because patients were investigated in only one region in Germany and not within a multicentred, European study, the results cannot be generalised, and they should be compared with caution to other countries with differing health care systems.

Overall, in terms of early detection and intervention, our results demonstrate the feasibility of implementing an external psychotherapeutic offering in the workplace in cooperation with the local occupational physicians and workplace health promotion programmes. Furthermore, the results of this study are valuable for tailoring a more extended intervention at this interface taking into account the needs of the patients and of the companies.

The key characteristic of a tailored intervention is that it should especially target the needs of the “at risk” group with interpersonal conflicts at the workplace. Moreover, the interface concerning referral into the existing secondary treatment system should be elaborated. Therefore, a stepped-care approach such as, for example, IAPT (Improving Access to Psychological Therapy) on a community-based level in the UK, appears promising [26].

After the user profiles have been identified, and it has been shown that the new offering addresses users early in the course of mental ill-health, we could show that PSIW is as effective as PSOC. These data were analysed as part of the study in a controlled observational trial, and are published elsewhere [55]. Further, the ingredients and interactions of the complex intervention PSIW must be investigated in detail. Our findings strongly support the need for mental health care specialist consultation in strong cooperation with the occupational physician or other company-based offerings. This subject is in ongoing analysis in a qualitative approach (grounded theory method).

## Supporting Information

S1 File Commented STROBE Checklist. (PDF)

S2 File Ethical Approval (Original). (PDF)

S3 File Ethical Approval (Translation). (PDF)

S4 File Funding statement Junior Academy Health Care Research Baden-Wuerttemberg. (PDF)

## Abbreviations

aBIC: adjusted Bayesian information criterion
AIC: Akaike information criterion
BIC: Bayesian information criterion
BLRT: bootstrap likelihood ration test
CLPA: conditional latent profile analysis
CMD: Common mental disorder
DP: Depersonalisation
DRKS: German Clinical Trials Register
EE: Emotional exhaustion
EM: expectation-maximisation
GP: General practitioner
IAPT: improving access to psychological therapy
IS: Irritation scale
LCA: latent class analysis
LPA: Latent profile analysis
M: Mean
MBI: Maslach burnout inventory
N: Number
n.s.: Not significant
OECD: Organization for Economic Cooperation and Development
PA: personal accomplishment
PHQ-7/-9/-15: Patient health questionnaire 7 items generalised anxiety/9 items depression/15 items somatoform symptom severity
PRO: patient reported outcome
PSIW: Psychotherapeutic consultation in the workplace
PSOC: Psychotherapeutic outpatient care
QoL: Quality of life
SD: Standard deviation
SF-12 mcs/pcs: Short form-12 Items mental health component score/physical health component score
STROBE: Strenghtening the reporting of observational studies in epidemiology
WA: Work ability
WAI: Work ability index
