# Supplementary material for: Improving Access to Mental Health Care by Delivering Psychotherapeutic Care in the Workplace: A Cross-Sectional Exploratory Trial
Source: PLoS One. 2017 Jan 5;12(1):e0169559. doi: 10.1371/journal.pone.0169559 (PMC5215922; doi:10.1371/journal.pone.0169559)
Supplement: S1 File — (PDF) [file pone.0169559.s001.pdf]

STROBE Statement: Checklist of items that should be included in reports of observational studies. Checklist annotated according to the manuscript, **"Improving access to mental health care by delivering psychosomatic consultation within the workplace: A cross-sectional exploratory trial".**

	Item No	Recommendation	Location in primary paper, or other details
**Title and abstract**	1	(*a*) Indicate the study's design with a commonly used term in the title or the abstract	*Done in the respective section ("title") of the manuscript*
		(*b*) In the abstract, provide an informative and balanced summary of what was done and what was found	*Done in the respective section ("abstract") of the manuscript*
**Introduction**			
Background/rationale	2	Explain the scientific background and rationale for the investigation being reported	*Done in: "Introduction"*
Objectives	3	State specific objectives, including any pre-specified hypotheses	*Done in: "Introduction", last paragraph. No pre-specified hypothesis, but "research question"*
**Methods**			
Study design	4	Present the key elements of the study design early in the paper	*Done in the first paragraph of "Methods", further detailed information in published "Study Protocol" (enclosed)*
Setting	5	Describe the setting, locations, and relevant dates, including periods of recruitment, exposure, follow-up, and data collection	*Setting and location is described under "Methods, Setting" Periods of recruitment are reported under "Methods, Participants". There is no follow up in this cross-sectional study. Data collection is described under "Methods, Measures"*
Participants	6	(*a*) *Cohort study*-Give the eligibility criteria, and the sources and methods of selection of participants. Describe methods of follow-up<br>*Case-control study*-Give the eligibility criteria and the sources and methods of case ascertainment and control selection. Give the rationale for the choice of cases and controls<br>*Cross-sectional study*-Give the eligibility criteria and the sources and methods of selection of participants	*Cross-sectional study: all criteria are reported in the respective section*
		(*b*) *Cohort study*-For matched studies, give matching criteria and number of exposed and unexposed<br>*Case-control study*-For matched studies, give matching criteria and the number of controls per case	--
Variables	7	Clearly define all outcomes, exposures, predictors, potential confounders, and	*Compare: Fig 1-Conditional latent profile model*

		effect modifiers. Give diagnostic criteria, if applicable	
Data sources/ measurement	8*	For each variable of interest, give sources of data and details of methods of assessment (measurement). Describe comparability of assessment methods if there is more than one group	*All variables are explained in "Methods", please also compare: the legend of Table 3: Latent profiles of impairment for four four-class solution*
Bias	9	Describe any efforts to address potential sources of bias	*Done in: "Methods, Bias"*
Study size	10	Explain how the study size was arrived at	*Done in: "Methods, Study size"*
Quantitative variables	11	Explain how quantitative variables were handled in the analyses. If applicable, describe which groupings were chosen and why	*Compare: Fig 1-Conditional latent profile model*
Statistical methods	12	(*a*) Describe all statistical methods, including those used to control for confounding	*Done in: "Methods, Analysis"*
		(*b*) Describe any methods used to examine subgroups and interactions	*Not applicable*
		(*c*) Explain how missing data were addressed	*Missing data were handled with full information maximum likelihood (FIML) estimation, as foreseen in MPlus*
		(*d*) *Cohort study*-If applicable, explain how loss to follow-up was addressed<br>*Case-control study*-If applicable, explain how matching of cases and controls was addressed<br>*Cross-sectional study*-If applicable, describe analytical methods while taking account of sampling strategy	*Not applicable*
		(*e*) Describe any sensitivity analyses	*Not applicable*
**Results**			
Participants	13*	(a) Report numbers of individuals at each stage of study, e.g. numbers potentially eligible, examined for eligibility, confirmed eligible, included in the study, completing follow-up, and analysed	*Done in "Participants" as applicable*
		(b) Give reasons for non-participation at each stage	*Not systematically assessed*
		(c) Consider use of a flow diagram	*See: Fig 2-Flowchart of participants*
Descriptive data	14*	(a) Give characteristics of study participants (e.g., demographic, clinical, social) and information on exposures and potential confounders	*Detailed in Table 1*
		(b) Indicate number of participants with missing data for each variable of interest	*See: Table 3-Latent profiles of impairment for four four-class solution. Numbers of complete cases per variable of interest are reported in Fig 2-Flowchart of participants*
		(c) *Cohort study*-Summarise follow-up time (e.g., average and total amount)	--
Outcome data	15*	*Cohort study*-Report numbers of outcome events or summary measures over	--

		time	
		Case-control study-Report numbers in each exposure category, or summary measures of exposure	--
		Cross-sectional study-Report numbers of outcome events or summary measures	*See: Table 1-Sample description, Fig 2-Flowchart of participants, and Table 5-Descriptive data of the four profiles*
Main results	16	(*a*) Give unadjusted estimates and, if applicable, confounder-adjusted estimates and their precision (e.g., 95% confidence interval). Make clear which confounders were adjusted for and why they were included	*See: Table 3-Latent profiles of impairment for four four-class solution*
		(*b*) Report category boundaries when continuous variables were categorised	*See: Legend Table 3-Latent profiles of impairment for four four-class solution*
		(*c*) If relevant, consider translating estimates of relative risk into absolute risk for a meaningful time period	*Not applicable*
Other analyses	17	Report other analyses done, e.g. analyses of subgroups and interactions, and sensitivity analyses	*Model fit parameters are reported (Table 2-Goodness of fit statistics for three to five class solutions)*
**Discussion**			
Key results	18	Summarise key results with reference to study objectives	*First paragraph of "Discussion"*
Limitations	19	Discuss limitations of the study, taking into account sources of potential bias or imprecision. Discuss both direction and magnitude of any potential bias	*Fifth paragraph of "Discussion"*
Interpretation	20	Give a cautious overall interpretation of results considering objectives, limitations, multiplicity of analyses, results from similar studies, and other relevant evidence	*Paragraphs 2–4 of "Discussion"*
Generalisability	21	Discuss the generalisability (external validity) of the study results	*End of paragraph five of "Discussion"*
**Other information**			
Funding	22	Give the source of funding and the role of the funders for the present study and, if applicable, for the original study on which the present article is based	*Details after "Discussion"*

*Give information separately for cases and controls in case-control studies and, if applicable, for exposed and unexposed groups in cohort and cross-sectional studies.

Note: An Explanation and Elaboration article discusses each checklist item and gives the methodological background and published examples of transparent reporting. The STROBE checklist is best used in conjunction with this article (freely available on the Web sites of PLoS Medicine at http://www.plosmedicine.org/, Annals of Internal Medicine at http://www.annals.org/, and Epidemiology at http://www.epidem.com/). Information on the STROBE Initiative is available at www.strobe-statement.org.
