# Supplementary material for: Improving Access to Mental Health Care by Delivering Psychotherapeutic Care in the Workplace: A Cross-Sectional Exploratory Trial
Source: PLoS One. 2017 Jan 5;12(1):e0169559. doi: 10.1371/journal.pone.0169559 (PMC5215922; doi:10.1371/journal.pone.0169559)
Supplement: S2 File — (PDF) [file pone.0169559.s002.pdf]

Gr. Rohmann

26. SEP. 2011

ulm university universität uulm

Ethikkommission

Universität Ulm • Ethikkommission • 89069 Ulm

Vorsitz: Prof. Dr. U.B. Brückner
Geschäftsstelle: Iris Seitz

Hausadresse:
Helmholtzstraße 20 (Oberer Eselsberg)
89081 Ulm
Telefon: +49 - (0)731 - 500-22052
Telefax: +49 - (0)731 - 500-22036
Email: ethik-kommission@uni-ulm.de
http://www.uni-ulm.de/ethikkommission/

Herrn
Prof. Dr. Harald Gündel
Klinik für Psychosomatische Medizin
und Psychotherapie
Am Hochsträß 8
89081 Ulm

Unser Zeichen	Durchwahl	Datum
224/11 – UBB/se.	22052	26.09.2011

n/an die Mitglieder der Ethikkommission der Universität Ulm

Antrag Nr. 224/11 – Die Psychosomatische Sprechstunde im Betrieb – ein neues Versorgungsmodell an der Schnittstelle zwischen betriebsärztlicher Betreuung und Konsiliarpsychosomatik

Sehr geehrter Herr Prof. Gündel,

am 05.09.2011 wurde Ihr o.g. Antrag mündlich verhandelt.

Nach Eingang der gewünschten Ergänzungen und Korrekturen am 23.09.2011 sind alle Voraussetzungen erfüllt.

Es bestehen keine ethisch begründbaren Bedenken gegen die Durchführung des Projekts.

Damit wird die Bewertung durch die Ethikkommission der Universität Ulm mit einer zustimmenden Stellungnahme abgeschlossen.

Entsprechend der ausschließlich beratenden Funktion der Ethikkommission betrifft diese Bewertung nur die *berufsethische und berufsrechtliche Beurteilung* der Konzeption, der geplanten Methoden, der Durchführung und Überwachung des betreffenden Projekts sowie der beabsichtigten Patientenaufklärung.

Die ärztliche und juristische Verantwortung verbleibt uneingeschränkt beim Projektleiter und den daran Mitwirkenden.

- Bitte teilen Sie uns jede Änderung in der Konzeption des Projekts mit. Es muss dann geklärt werden, ob die Beurteilung der Ethikkommission noch Bestand hat.
- Über alle schwerwiegenden oder unerwarteten unerwünschten Ereignisse, die während der Untersuchung auftreten und die Sicherheit der Studienteilnehmer oder die Durchführung des Projekts beeinträchtigen könnten, muss der Vorsitzende der Ethikkommission unterrichtet werden.
- Die Ethikkommission der Universität Ulm geht davon aus, dass nicht-ärztliche Mitarbeiter speziell auf Verschwiegenheit verpflichtet werden.
- Außerdem benötigt die Ethikkommission eine zeitnahe Nachricht über den Abschluss des Projekts und einen Bericht mit Mitteilung der gewonnenen Erkenntnisse.

Für die Ethikkommission der Universität Ulm

Prof. Dr. U. B. Brückner
Vorsitzender

Mitglieder der Kommission: Prof. Dr. U.B. Brückner (Vorsitz), J. Glembek, Prof. Dr. J. Högel, Prof. Dr. P. Kern, PD Dr. M. Kölch, Dr. iur. H.-D. Lippert, Pfarrer E. Schäfer, Prof. Dr. H. Schrezenmeier, Frau Prof. Dr. J. Stingl, Frau Prof. Dr. H. Suger-Wiedeck
