# Supplementary material for: Improving Access to Mental Health Care by Delivering Psychotherapeutic Care in the Workplace: A Cross-Sectional Exploratory Trial
Source: PLoS One. 2017 Jan 5;12(1):e0169559. doi: 10.1371/journal.pone.0169559 (PMC5215922; doi:10.1371/journal.pone.0169559)
Supplement: S4 File — (PDF) [file pone.0169559.s004.pdf]

UniversitätsKlinikum Heidelberg
Koordinierungsstelle Versorgungsforschung Baden-Württemberg
Abteilung Allgemeinmedizin u. Versorgungsforschung
Voßstr. 2, Geb. 37
D-69115 Heidelberg

Eva Rothermund
Universität Ulm
Klinik für Psychosomatische Medizin und Psychotherapie
Am Hochsträss 8
89081 Ulm

[redacted]
Koordinierungsstelle Versorgungs-
forschung Baden-Württemberg

PD. Dr. Gunter Laux
Abteilung Allgemeinmedizin
und Versorgungsforschung
Voßstraße 2, Geb. 37
D-69115 Heidelberg
Fon +49 (0)6221 56 6207
Fax +49 (0)6221 56 1972
g.laux@med.uni-heidelberg.de

Heidelberg, 9.9.2011

**Ihre Förderung im Rahmen der
Nachwuchsakademie Versorgungsforschnnug Baden-Württemberg**

Sehr geehrte Frau Rothermund,

wir freuen uns sehr, dass Sie mit Ihrem Forschungsprojekt die erforderlichen Bedingungen zur Förderung im Rahmen der Nachwuchsakademie Versorgungsforschung Baden-Württemberg erfüllt haben. Sämtliche Informationen bzgl. Ihrer Förderung finden Sie anbei.

Bitte teilen Sie uns noch folgende Informationen mit, die für eine Mittelzuweisung notwendig sind:

Angabe eines deutschen **Bankkontos**, auf das die Mittel an Ihr Forschungsinstitut überwiesen werden, mit Angabe von

- Name des Kreditinstituts
- Bankleitzahl
- Kontonummer

<u>und</u>

sofern vorhanden, die Angabe der **Kostenstelle**, über die Ihr Forschungsprojekt geführt wird. Diese Kostenstelle erscheint als Verwendungszweck bei den jeweiligen Überweisungen.

Bitte senden Sie diese Angaben per Email an den Kostenstellenverantwortlichen unserer Abteilung, Herrn Andreas Gutscher, der Ihre Angaben diskret behandeln wird.

Email-Adresse: andreas.gutscher@med.uni-heidelberg.de

Mit freundlichen Grüßen,

Heidelberg, den 9.9.2011 ______________________
(PD Dr. Gunter Laux)

[redacted]
