# Supplementary material for: Improving Access to Mental Health Care by Delivering Psychotherapeutic Care in the Workplace: A Cross-Sectional Exploratory Trial
Source: PLoS One. 2017 Jan 5;12(1):e0169559. doi: 10.1371/journal.pone.0169559 (PMC5215922; doi:10.1371/journal.pone.0169559)
Supplement: S3 File — (PDF) [file pone.0169559.s003.pdf]

[ulm university – universitaet logo]
[uulm]

[date stamp] September 26, 2011

Ethics Committee

University Ulm   Ethics Committee   89069 Ulm

Mr.
Prof. Dr. Harald Guendel
Clinic for Psychosomatic Medicine and Psychotherapy
Am Hochstraess 8
89081 Ulm

Chairman: Prof. Dr. U. B. Brueckner
Main office: Iris Seitz
Address:
Helmholtzstrasse 20 (Oberer Eselsberg)
89081 Ulm
Phone: +49 – (0)731 – 500-22052
Fax: +49 – (0)731 – 500-22036
Email: ethic-kommission@uni-ulm.de
http://www.uni-ulm.de/ethikkommission/

Our Signature	Direct line	Date
224/11 – UBB/se.	22052	09.26.2011

n/To the members of the Ethics Committee of the University of Ulm

Proposal Nr. 224/11 – The psychosomatic consultation at the workplace – a new model of care at the interface of company-supported mental health care and consultation-liaison psychosomatics

Dear Prof. Guendel,

On 09/05/2011, we started to negotiate your proposal. After all required adjustments were made on 09/23/2011, we no longer find any ethical concerns in the implementation of your project.

The Ethics Committee of the University of Ulm approves the above referenced research.

According to the advisory function of the Ethics Committee, this assessment only pertains to the ethical and professional rules and assessment of the research concept, the proposed methods, the implementation and monitoring of the project as well as patient informed consent.

All medical and legal responsibility remains with the project manager and project staff.

- Changes in the research or the informed consent process must be approved by the Ethics Committee prior to implementation.
- The chairman of the Ethics Committee needs to be informed promptly in the event that serious, unexpected and related adverse events occur during the investigation which could affect the safety of study participants or the safe execution of the project.
- The Ethics Committee of the University of Ulm presumes that non-medical project staff are committed to confidentiality.
- The Ethics Committee requires a timely notification about the project's completion and a report about its findings.

For the Ethics Committee of the University of Ulm

[signature]
Prof. Dr. U. B. Brueckner
Chairman

Members of the Committee: Prof. Dr. U.B. Brückner (Chairman), Prof. Dr. J. Högel, prof. Dr. P. Kern, PD; Dr. M. Koelch, Dr. jur. H.-D. Lippert, Pastor E. Schaefer, Prof. Dr. H. Schrezenmeier, Mrs. Prof. Dr. J. Stingl, Mrs. Prof. Dr. H. Suger-Wiedeck
